# Breaking the scattering limits of water waves

**DOI:** 10.1093/nsr/nwae138

**Published:** 2024-05-02

**Authors:** Adrià Canós Valero, Thomas Weiss, Yuri Kivshar

**Affiliations:** Institute of Physics, University of Graz, Austria; Institute of Physics, University of Graz, Austria; 4th Physics Institute, University of Stuttgart, Germany; Research School of Physics, Australian National University, Australia

## Abstract

Discover how breakthroughs in metamaterials can reshape ocean engineering, creating water mirages with the help of carefully designed obstacles.

When water, light or sound waves impinge on objects smaller than the wavelength, a scattering process takes place [1]. The scattering of waves plays an essential role in a myriad of natural phenomena. In ocean engineering, enhancing the scattering of water waves allows capturing energy from larger areas, with clear implications for energy harvesting and coastal protection.

However, there exists a general constraint to the maximum scattering by small objects, ‘scatterers,’ known as the ‘single channel limit’ [1,2]. Recently, however, a series of pioneering works in metamaterials have demonstrated that such seemingly strict bounds can be exceeded by a careful design of the geometrical degrees of freedom of the scatterer, forming the so-called ‘superscatterer’ [1–4]. This boosts the scattering cross-section significantly, creating the illusion of a much larger object.

The initial experimental confirmation of superscattering with electromagnetic waves occurred only a few years ago in the microwave regime [2]. Despite existing technical challenges, the potential applications are already promising, including enhanced capabilities in energy harvesting, sensing, and target tracking. Importantly, superscattering is not limited to electromagnetism; it is a general wave phenomenon expected to manifest in other areas of wave physics.

In an exciting breakthrough, the authors of Ref. [5] have now predicted and demonstrated superscattering with water waves. Their work opens new avenues for ocean engineering and expands the scope of applications for superscatterers.

The authors modeled linear, inviscid water waves on the surface of a large water tank with a submerged cylindrical scatterer. These approximations enabled direct analogies between electromagnetic theory and fluid dynamics. In electromagnetic theory, superscatterers are usually created by stacking concentric layers with different refractive indices. In fluid dynamics, the refractive index role is played by the height of each layer in the cylinder, thereby modulating the water levels (Fig. 1b, top).

**Figure 1.**
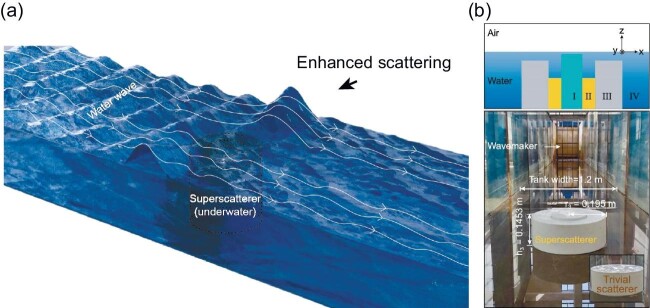
Tricking the waves to grow tall or shrink down. (a) Concept of superscattering of water waves. (b) Top: sketch of the design. Bottom: experimental realization. Adapted with permission from Ref. [5].

The designed scatterer yielded a scattering cross section over three times the single channel limit. This led to strong interference between the scattered and incident waves, forming areas with large vertical water displacements and ‘calm’ regions with minimal disturbances (Fig. 1a). Analytical and numerical simulations validated these predictions.

To experimentally observe the effect, the authors 3D-printed a conventional scatterer and a superscatterer of identical size, placing them separately in finite water tanks (Fig. 1b, bottom). Incident water waves were employed to probe their individual responses, revealing a significantly larger field distortion for the superscatterer compared to the conventional object. The manifestation of superscattering was illustrated by introducing a plastic boat near the superscatterer, demonstrating amplified or suppressed vertical motion depending on their relative positions.

While this achievement is a great first step, there is still a long road ahead. First, as the authors also acknowledge, it will be interesting to understand whether superscattering can still occur in the presence of nonlinearities in the water waves, as well as viscosity. Second, more complex shapes besides cylindrical objects can be investigated.

As metamaterials venture into these uncharted waters, we cannot wait to see what will come next.
